# A study on visual preference for social stimuli in typical Ecuadorian preschoolers as a contribution to the identification of autism risk factors

**DOI:** 10.1038/s41598-021-87888-3

**Published:** 2021-04-19

**Authors:** Paulina Buffle, Thalia Cavadini, Andres Posada, Edouard Gentaz

**Affiliations:** 1 Faculty of Psychology and Educational Sciences, Department of Psychology, University of Geneva, 40 boulevard du Pont-d’Arve, 1211 Geneva 4, Switzerland; 2 Swiss Center for Affective Sciences, University of Geneva, Geneva, Switzerland; 3 CNRS, Grenoble, France

**Keywords:** Human behaviour, Social behaviour

## Abstract

The goal of this study was to examine the visual preference towards socially salient stimuli, using a low-cost eye-tracking device in a group of typically developing (TD) Ecuadorian preschoolers aged 11 to 60 months, from rural and urban areas, and from families with low to high socioeconomic status (SES). Series of original stimuli inspired by those used in Western experiments on the early detection of Autism Spectrum Disorder (ASD) were proposed in two eye-tracking tasks. Two types of movements (human vs. object) were presented in task 1, and dynamic speaking faces in task 2. Parental perceptions of the adaptability of the low-cost eye-tracking device used here were also investigated through a questionnaire. The analyses of mean fixation times showed a visual preference for human movements compared to moving objects whatever age, residency location or SES. In task 2, visual preference for the mouth’s area compared to the eyes’ area was observed in specific conditions, modulated by residency location and SES but not by age. The analyses of the parental perception indicated that the eye-tracking technique is well accepted. The findings suggest that these stimuli, along with the experimental procedure and low-cost eye-tracking device used in the present study may be a relevant tool that can be used in clinical settings as a contribution to the early identification of at-risk factors of ASD in low- and middle-income contexts.

## Introduction

One of the major global child health challenges of the twenty-first century is the early identification of children with a developmental condition such as Autism Spectrum Disorder (ASD). ASD refers to a group of complex neurodevelopment conditions with an estimated prevalence of at least one percent for the broad autism spectrum. ASD is characterized by atypical social communication and interaction, and by repetitive patterns of behaviors^1^. The necessity of early identification of risk factors is reinforced by a recent understanding of brain plasticity during the first years of development^2^. Etiologic investigation strongly supports a genetic-environment effect^3–5^. Different research teams in highly resourced laboratories working with various techniques, such as molecular approaches, sequencing, fMRI, and MEG, have made considerable efforts to provide explanations of the behavioral phenotype. So far, a reliable biomarker has not yet been identified^6^. Other investigations have been focusing on the behavioral socio-cognitive variables such as the perceptual-cognitive mechanisms that play a role in the identification of relevant social information, for example, the emotional content of faces^7^, and the ability of spontaneously turning to other people^8–10^. These behavioral markers, which are currently used in ASD diagnostic and screening procedures, could suffer from the influence of parental knowledge on child development and culturally defined expectations of child behavior.

Standardized assessment procedures applicable across all socio-cultural contexts are critical for early identification. Hence, local lines of research should be stimulated and complementary approaches to clinical observation need to be explored. Eye-tracking is a promising technology for the study of social orientation behaviors that has the potential to be adapted to different socio-cultural contexts^11,12^. It allows the use of standardized procedures and provides measures reported to be valid and particularly well suited to the study of pre-verbal populations^13^. Few studies examined the ocular movements with an eye-tracking device in infants, even though its spatial and temporal precision and accuracy allow us to calculate precisely the time and the direction of the gaze. In addition, the cost of such devices can be affordable, as companies have proved feasible the production of eye-tracking under USD 100, complemented with open source software for the data analysis. This non-invasive technique has the potential to identify markers of neurocognitive and social development^14^.

Based on the hypothesis that a gaze fixation on a specific point provides a measure of attention^15,16^, eye-tracking technology has been used for the study of visual attention to human movements with a variety of stimuli, such as light points^17^. However, the use of this technology also depends on the age of participants. With very young infants, behavioral studies usually use the videos in individual testing sessions requiring the experimenters to manually code the infant’s gaze as being directed either to the left side, the right side, or outside of the screen, generating raw looking data. Using this classic coding, studies showed orientation towards human movements is an early ability present in neonates^18,19^, and this precocity has been described in other species^20^. Studies using human actors have also suggested that orientation towards biological movements is a key determinant of typical development (TD) and might serve as an indicator of the development of social cognitive skills. Using eye-tracking technology, a study performed with three groups of children (37 with ASD, 22 with developmental delay, and 51 typically developing toddlers), of 12 and 42 months of age presenting a one-minute video with socially salient scenes with moving children (gymnastics, yoga, dance) on one side of the screen and geometric figures in motion on the other side, has indicated that, when young children have a choice, they prefer to look at social images rather than non-social images^21^. The reduction in orientation towards dynamic social images (children moving) and more time spent on moving geometric shapes characterized the group of toddlers with ASD. Using a similar paradigm with an eye tracking technology, Franchini et al.^22^ replicated those results in a group of 33 school-aged participants with ASD who oriented less towards dynamic social scenes compared to the TD control group.

The eye-tracking technique has also been used in the study of the socio-cognitive processes that sustain more complex abilities, such as visual attention to faces. The preference to faces plays an important role in human development, allowing the adaptation of the individual to his/her environment, as well as in the engagement with the social world, to survive and to learn from others^8,23,24^. Reduced attention to others negatively affects the ability to join in imitative games and reciprocal vocalizations^25,26^. This attentional process is highly conserved phylogenetically^27^, and appears very soon after birth, with a specific attentional focus towards the eyes^28^. For example, using a classic coding, the visual fixations of 3- to 5-week-old, 7-week-old, and 9- to 11-week-old infants were analyzed as they scanned an adult's face who was stationary, moving, or talking^29^. The authors observed a dramatic increase in face fixations between 5 and 7 weeks in all conditions and an intensification of scanning in the eyes’ area in the two oldest groups in the talking condition. Few studies examined the specific areas of interest (AOIs) on a face stimulus (e.g. the eyes and mouth) in typically developing infants using an eye-tracking device. For example, Dollion et al.^30^ using eye-tracking and dynamic emotional faces with infants aged from 3 to 12 months of age have shown that younger infants focused their attention to the eyes and external features of emotional faces whereas the attention of older infants (7 and 12 months of age) depended on the emotion presented. Mouth gets the attention in smiling faces, eyes and eyebrows in fearful and angry faces, and the upper nose area in disgusted faces. By contrast, Hunnius et al.^31^ have shown that 4- and 7-month-old infants looked longer at the mouth of angry faces. More recently, Palama e al.^32^ observed the absence of significant spontaneous visual preferences for either one of the two static emotional faces (of joy and anger) presented simultaneously, nor for specific areas in 6-month-old infants. Using eye-tracking technology, visual orientation towards faces appears to be reduced in toddlers and preschoolers with ASD compared to typically developing peers^33^ and visual fixation towards the eyes has been reported to be significantly impaired in 2-year-olds with ASD^34^. Using a naturalistic scene of a caregiver addressing participants, Jones and Klin^35^ reported that in infants later diagnosed with ASD, preferential attention to the eyes declines from 2 months of age, showing a level that is about half that of typically developing children by 24 months of age. This study also indicated that the fixation times on the mouth’s area starts to increase from 2 months until approximately 18 months of age. However, other findings suggested that reduced fixation times on the eyes may not be present in toddlers with ASD: Chawarska and Shic^36^ conducted a longitudinal study with ASD and TD children aged 2 years and observed similar fixation times on the eyes’ area in both groups. Authors also indicated that, at 4 years of age, children with ASD spend less time looking at the eyes, mouth, and nose areas than typically developing children, but the difference in the amount of fixation on the eyes between groups was not significant.

Together, those findings suggest that the typical visual preference patterns towards socially salient stimuli involve, comprehensively, an increasing orientation towards human movements versus object movements and an increasing orientation towards the eyes’ area, a decreased orientation towards the mouth. The opposite pattern could be considered as a reliable quantifier of social disability^37^.

Mixed findings may result from different factors such as the type of stimuli and the analysis techniques used, which allows studying social orientation under a variety of conditions but limits cross-study results comparisons^38^. Cross-cultural comparisons are also limited, as the eye-tracking technique has been essentially used in Europe, North America, and Japan and mainly in highly resourced academic settings. Studies from diverse cultural settings are scarce, particularly in preschool-age children. Some of these studies on visual preferences towards socially salient stimuli in Asian countries reported an impact of cultural context on the way humans look at faces. For example, Liu et al.^39^ indicated a preference for the central part of a face (nose region) in 9-month-old Asian infants. Another study that compared Western Caucasian and East Asian 7–12 years old children suggested that individuals from Asian cultures do not preferentially fixate the eyes’ region of the face, directing instead fixations on the nose^40^. The same conclusions are reached when comparing Western Caucasian and East Asian adults. These eye-tracking findings are coherent with the cultural expectations that direct eye contact is considered rude in many Asian cultures.

Studies on visual orientation towards social stimuli are not reported in Latin American countries. Henceforth, there is a need to investigate the contribution of a low-cost eye-tracking display to identify at-risk factors of ASD in low- and middle-income settings, such as rural clinical practices and community services. Socio-cultural factors, together with other variables, such as unfamiliarity with test situations and with screen-based tools, particularly in the case of eye-tracking, could also influence the visual fixation results and the successful implementation of assessment techniques in culturally diverse settings.

### Objectives and hypotheses

The main objective was to examine the visual preferences towards socially salient stimuli using a low-cost eye-tracking device in a group of typically developing Ecuadorian preschoolers from rural or urban areas and from families with low to high socioeconomic status (SES). Series of original stimuli inspired by those used in Western experiments on the early detection of ASD were proposed to examine visual preferences in two tasks. Two types of movements (human vs. object) were presented in task 1 and inner dynamic talking faces were presented in task 2. Parental perceptions of the adaptability of the eye-tracking setting were investigated through a short questionnaire.

We hypothesized that a visual preference for human movements compared to object movements in task 1 and for eyes compared to mouth should be observed. We also investigated whether these visual preferences are modulated by age, residency location or SES factors. Finally, we hypothesized that the eye-tracking setting would be well accepted and adapted to study child development in a non-academic context in a low- and middle-income contexts.

## Methods

### Participants

A final sample of 37 typically developing children (aged from 15.2 to 59 months; *M* = 33.37, *SD* = 15.22) reached the inclusion criteria for eye-tracking tasks. Participants were recruited through public announcements in the town of Quito and its rural surroundings. The absence of developmental disorders in these children was controlled with a questionnaire addressed to parents. Children younger than 31 months were assessed by the Spanish version of M-chat, validated for a Mexican population^41^. Children older than 32 months were assessed with the SCQs translated to Spanish^42^. Both instruments were self-administered by the primary caregivers, assisted by the researcher when requested. The Bayley Scales of Development was used to assess the level of communication in children younger than 42 months. The VABS^43^ was used to assess the scale of language on children older than 42 months. Information on hearing and visual status, considered to be a factor of exclusion in cases of impairment or deficit in this study, was collected through a parental questionnaire.

Parents were also interviewed using a socio-demographic questionnaire. These socio-demographic characteristics are described in Table 1.

**Table 1:** Parents socio-demographics characteristics.

	Both tasks	Task 1: human vs. object movements	Task 2: mouth region vs. eyes region
	*N* = 37	*N* = 30	*N* = 27
Mean age (months)	33.3 (± 12.20)	33.1 (± 12.09)	33.1 (± 10.86)
Age min months	15.22	15.22	15.22
Age max	59	59	57.13
**Gender**			
Male	19	15	10
Female	18	15	17
**Socioeconomic Status**^**a**^			
Medium–high to high (HMH)	21	14	14
Low to medium–low (LML)	16	16	13
**Residency Area**			
Urban	19	15	16
Rural	18	15	11
**Parents**			
Mean age fathers	30.32	30.56	29.70
Mean age mothers	28.78	28.87	28.25
*Level education fathers*			
Post-secondary	23	17	17
Secondary	13	12	10
Primary	1	1	0
*Level education mothers*			
Post-secondary	20	13	15
Secondary	15	15	11
Primary	2	2	1

^a^The family’s socioeconomic status (SES) was approximated using the price of the preschool as a proxy ranging from one to two (the highest SES).

Parents gave their informed written consent for their child to take part in the present study, which was conducted in accordance with the Declaration of Helsinki and whose experimental protocol had been approved by the Ethics Committee of the University of Geneva. Parents of acting children and adults playing the role of a “caregiver,” provided an informed written consent for participation and for publication on an online open-access publication.

### Experimental procedure

Children accompanied by a parent were hosted either in a separate room provided by the city's playschools or in a communal house in rural areas. In both cases, the experiment took place in a closed room where the light could be controlled and adapted to the optimal conditions for the eye-tracking setting. Each child was tested individually. Children were placed on an adjustable height seat with a headrest behind their neck to maintain head stability. Parents stood behind the children and were required not to move for the duration of the experiment in order to avoid distracting or moving the child or having their eyes captured by the eye-tracker. Children were seated about 60 cm from a device (Dell screen of 47.5 × 30 cm with a spatial resolution of 1680 × 1050 pixels) equipped with an “EyeTribe” (https://theeyetribe.com/theeyetribe.com/about/index.html) eye-tracker (an adjustable table was used to set the height of the stimulation screen so that the participant's eyes were positioned in the center of the screen when looking straight ahead as recommended). Eye-tracking settings, such as this one, provide non-invasive measurement of fixation times using corneal reflection to detect oculomotor movements. The system records near-infrared reflections of both eyes at 60 Hz (accuracy = 0.5°, spatial resolution = 0.1°). Accuracy and precision of outcomes for image-based studies comparable to those of well-established eye-tracking devices^44,45^, and it is considered to be a valuable resource for research in cognitive psychology^12,46^. The eye-tracker was positioned at approximately 45 cm from the child's face, ensuring that its top side was aligned with the underside of the stimulation screen^45^.

The experiment consisted of four blocks: a first calibration phase followed by two visual preference tasks (randomly ordered) and by a short parental questionnaire to investigate parents’ perception of the experiment. A calibration with 9 points was required to verify that the eye motion could be directed to different areas. Quality of calibration was displayed on the screen. If a child’s calibration was poor, the procedure was repeated as necessary. Participants were required to look to the circular target that was displayed at different locations of the screen on a blank background, for approximately 2 s each. This phase was repeated until a satisfactory calibration was achieved: it was only once the eye-tracking device was successfully calibrated that the two visual preference tasks (lasting approx. 35 s and about 1 min each) could begin. Both tasks were designed to test the tendency to look longer (in terms of mean fixation times) at socially salient stimuli. The order of presentation of these two tasks was balanced to limit a possible drop in attention (order effect). At the beginning of each task, a children's character with a music-box sound was presented for 4 s in order to bring the children's attention to the screen. The same character and sound had already been presented for 4 s at the very beginning of the experiment (before the calibration phase) for the same purpose. Both types of stimuli were recorded with the participation of South American actors and were previously tested in a population of 15 adults. These stimuli were judged as neutral to an Ecuadorian population.

Before leaving, the parents were quickly questioned about their perception of the experimental procedure that their child had just gone through, and the child received a wooden-puzzle (worth approx. USD 3) at the end of the experiment as a thank you for participating.

### Measures

#### Task 1: visual preference for human vs. object movements

**Figure 1:**
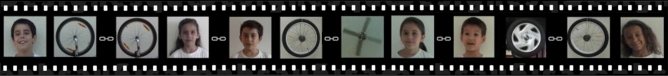
Serie of original stimuli used to quantify the visual preferences for two types of movements (human vs. object). Each pair of stimuli was presented for 5 s., for a total task duration of 34.5 s.

In the first task, we used a procedure inspired by the stimuli used by Pierce et al.^21^. In this study, authors projected, side by side, a series of pairs of images on a screen to children aged between 12 and 42 months with and without ASD. To study the visual preference for social versus non-social scenes, we used a paradigm of preferential fixation time, quantified by the mean fixation time on socially prominent scenes (faces of children in movement) compared to repetitive movements of the object. Although the preference for human movements cannot be fundamentally questioned when using a stimulus based on irregular movements across the screen, such as geometric motion used by Pierce et al.^21^, we assumed that the pattern of movements used to study the preference could affect the outcomes. For this reason, we created a stimulus using spinning objects that are centered. In this case the AOIs were designed to fill approximately the same proportion of space in the screen for human and for non-human (object) movements. Hence, only the head region of actors was shown to participants. Round objects were chosen for object movements aiming to make the comparison more precise. Social scenes were recorded in a neutral environment with white background and consisted of videos of children moving their heads, faces, and mouths naturally. Object movement scenes consisted of the repetitive movement of a bicycle wheel or a fan (cf. Figure 1 for screenshots or see videos 1 to 6 in online associated Supplementary material). The final stimulus was made up of six pairs of scenes distributed horizontally across the screen, each one measuring approximately 9 × 10 cm, that corresponded to the size of the AOIs, with a distance of 3 cm between the 2 stimuli and was presented on a dark grey background. The visual angles of the stimuli were Horizontal: 20°, Vertical: 10°. No sound accompanied the stimuli. The duration of the presentation of each pair of scenes was of 5 s. The presentation of human and object stimuli was counterbalanced between the left and right sides of the screen in order to control a spatial preference. The presentation of social stimuli was also alternated between male and female actors. The influence of low-level elements, such as the color of faces was not controlled, to maintain their ecological character. Likewise, the speed of movement of children's faces has been kept to preserve the natural character of these movements and may not correspond to the speed of movement of objects that were filmed and presented at their usual speed. As far as possible, the color temperatures on both sides of the screen were standardized. Between each sequence, we presented a turning wheel in the middle of a black background screen (approx. 2 × 2 cm) for 1.5 s., and after 3 sequences a character with a music-box sound was presented, aiming to bring participants’ attention back to its center. The total duration of the presentation of the sequences was of 34.5 s, with 25 frames per second.

#### Task 2: visual preference for the facial region of the mouth vs. the eyes

**Figure 2:**
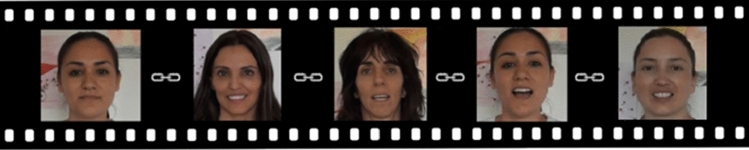
Serie of original stimuli used to quantify the visual preferences for the eyes’ region vs. the mouth’s region. The average duration of each of the five sequences was 11.5 s, for a total presentation time of approx. 1 min.

In the second task, we followed a procedure inspired by the studies of Jones, Carr and Klin^34^ and Jones and Klin^35^. The stimuli we designed consisted of five video-clips (available in online associated Supplementary material), each presenting a naturalistic scene of an actress addressing directly to the camera playing the role of a “caregiver” and singing nursery rhymes in Spanish, intended to attract the child's attention (Fig. 2). The actress was filmed in an ecological setting simulating a room with paintings in the background. The final stimulus was made up of 5 scenes distributed vertically across the screen, each one measuring approximately 16 × 13 cm and was presented on a dark grey background. The sizes of the 2 AOIs was 6.5 cm. × 3.5 cm, with a distance between AOIs of 4.5 on average depending on the size of the face. The visual angles of the stimuli were Horizontal: 15°,Vertical: 12°. The videos were projected for a total duration of just over 1 min, with 25 scenes per second. The sound was output through speakers not visible to the children. The audio duration was on average 11.5 s. The volume of auditory stimuli presented did not exceed 60 dBA.

#### Parental perception of the adaptability of eye-tracking to assess young children with a short questionnaire

In order to study the parent's perception of the eye-tracking as an assessment technique, parents were asked (1) if they felt comfortable during the experiment with their child, (2) if they would recommend a friend to participate in the assessment with their child^11^, and (3) if they had questions related to the experiment. The response options for the first two questions were “yes”, “no” and “maybe”.

### Data processing

All oculometric data (fixation times in milliseconds) were extracted using Open Gaze and Mouse Analyzer (Ogama—version 5.0), developed by researchers at the Free University of Berlin. In each of the two eye-tracking tasks, only data from participants who looked at the stimulation screen for more than 50% of the time were analyzed.

The first task was completed by 43 children (data from three participants were excluded due to procedural failures) but only 30 of them looked at the screen long enough during the task (more than 50% of the time) to include their data in our analyses. This sample (*N* = 30; 15 females) consisted of full-term born (at least 37 weeks of gestation) children aged 15.2 to 59.2 months (*M* = 32.7, *SD* = 10.3) divided into the following three age groups: 11–24 months (*n* = 8); 25–36 months (*n* = 11); 37–60 months (*n* = 11). Their family socioeconomic status (SES) was calculated as a proxy of the cost paid monthly by the preschool and classified in “low to medium–low (LML) SES” (*n* = 9) and “medium–high to high (HMH) SES” (*n* = 21). They were living in urban and rural areas (see Table 1 for more details).

Although our 46 participants completed the second task, only the data from the 27 children who spent more than 50% of the task duration looking at the stimulation screen were analyzed. The descriptive characteristics of this sample are as follows: children (*N* = 27; 16 females) aged 15.2 to 48.5 months (*M* = 32.4, *SD* = 9.7) were all born at least at 37 weeks of gestation. These children were distributed among three age groups as follows: 11–24 months (*n* = 6); 25–36 months (*n* = 12); 37–60 months (*n* = 9). They came from families with LML (*n* = 7) and HMH (*n* = 20) socio-economic status and were living in urban (*n* = 17) and rural (*n* = 10) areas (cf. Table 1).

### Statistical analysis

Statistical analyses were computed using TIBCO Statistica 13.2 computer software. We used an alpha level of 0.05 for all statistical tests.

Firstly, we implemented a non-factorial plan with two AOIs (human vs. object movements in task 1 and mouth vs. eyes’ areas in task 2) as independent variable. In the first task, AOIs corresponded to the size of each stimuli (i.e. 9 × 10 cm). In the second task, we defined two AOIs corresponding to the eye’s region and the mouth region, each measuring 6.5 × 3.5 cm, with a distance between the two AOIs of 4.5 cm on average (depending on the size of the face).

We aimed to analyze the mean fixation time, assuming that longer fixation duration is a measure of a more pronounced visual engagement. Focusing, at first, on the analysis of the mean fixation time of all the subjects (in average) towards the two AOIs, we conducted a *T*-test for independent samples. We then focused on the analysis of the mean fixation time within-subjects towards the two AOIs by performing a *T*-test for dependent samples in order to explore whether each participant looked at both AOIs equally.

Secondly, we conducted a series of factorial analyses to test the influence of the following three factors on the visual preference for socially salient stimuli: age group (11–24, 25–26 vs. 27–60 months), residency location (rural vs. urban areas), and SES (LML vs. HMH). For this purpose, three separate analyses were conducted: (1) a *T*-test for dependent samples was used to test whether the individuals in each group category thus formed looked significantly longer at one of the two stimuli presented simultaneously during the two tasks (intra-group difference), (2) inter-group comparisons were performed (using either a *T*-test for independent samples or an One-way ANOVA according to the number of factor modalities) to test whether individuals in the different group categories had equivalent or significantly different fixation times towards each stimulus, and (3) mixed factorial ANOVAs finally enabled us to test the interaction between the stimuli type/area (in task 2) and the group categories.

## Results

### Visual preference in task 1 (human vs. object movements)

The first result for the whole sample indicated that the average of the mean fixation time on human movements was significantly higher (*M* = 1683.67, *SD* = 390.11), compared to object movements (*M* = 774.85, *SD* = 394.65). Indeed, the analysis considering the mean fixation time of all the subjects toward each of the two AOIs independently was significant [*t*(58) = 8.97, *p* < 0.001] as well as the analysis of the mean fixation time within-subjects towards the two AOIs, *t*(29) = 6.987, *p* < 0.001.

We found similar results in our three factorial analyses: children in all three age groups also looked at human movements longer than at object movements (see intra-group difference in analysis b in Table 2), as do children living in rural and urban areas (see intra-group difference in analysis c in Table 2) and those from families with low to medium–low and medium–high to high SES (see intra-group difference in analysis d in Table 2).

**Table 2:** Mean (SD) fixation times in ms. as function of movement type, age group, residency location and SES.

	*N*	Fixation time (ms.)				Intra-group difference^1^	
		Human movements		Object movements		*t*	*p*
		*M*	(*SD*)	*M*	(*SD*)		
**(a) Total sample**	30	1683.67	(390.11)	774.85	(394.65)	6.987	< .001
**Factorial analysis**							
**(b) Age group**							
11–24 months	8	1783.96	(482.35)	601.53	(268.18)	4.662	0.002
25–36 months	11	1654.64	(449.18)	969.60	(494.33)	2.574	0.028
37–60 months	11	1639.76	(256.16)	706.15	(293.31)	6.651	< .001
Inter-group difference^2^	*F*(1, 27) = 0.348, *p* = 0.709			*F*(1, 27) = 2.516, *p* = 0.10			
Interaction^3^	*F*(1, 27) = 1.151, *p* = 0.331						
**(c) Residency location**							
Rural areas	14	1629.13	(235.55)	810.69	(252.93)	7.837	< .001
Urban areas	16	1731.40	(490.85)	743.49	(493.39)	4.325	< .001
Inter-group difference^4^	*t*(28) = -0.71, *p* = 0.483			*t*(28) = 0.459, *p* = 0.65			
Interaction^3^	*F*(1, 28) = 0.414, *p* = 0.525						
**(d) SES**							
LML	9	1748.20	(482.46)	661.82	(345.51)	4.137	0.003
HMH	21	1656.02	(353.39)	823.29	(412.16)	5.582	< .001
Inter-group difference^2^	*t*(28) = -0.586, *p* = 0.562			*t*(28) = 1.028, *p* = 0.313			
Interaction^3^	*F*(1, 28) = 0.793, *p* = 0.381						

Results of T-tests and ANOVAs about intra- and inter-group differences and interactions are indicated in each condition.

^1^T-test for dependent samples; ^2^One-Way ANOVA; ^3^Mixed factorial ANOVA;^4^T-test for independent samples.

Moreover, the different modalities of these factors did not seem to influence the visual preferences of the individuals they clustered: the results showed that there were no significant differences in the fixation times for each stimulus between each group category (see inter-group difference analyses in Table 2).

Finally, the interaction (stimulus type*group) was non-significant neither for the age group [*F*(1, 27) = 1.151, *p* = 0.331], residency location [*F*(1, 28) = 0.414, *p* = 0.525] nor SES [*F*(1, 28) = 0.793, *p* = 0.381].

### Visual preference in task 2 (mouth vs. eyes’ region)

The first *T*-test performed on the whole sample group’s mean fixation time on the two AOIs (mouth vs. eyes’ areas), indicated that the average of the mean fixation time on the region of the mouth (*M* = 3906.80, *SD* = 2187.18) was significantly higher than on the eyes (*M* = 2546.52, *SD* = 2056.86), *t*(52) = -2.354, *p* = 0.022. However, when intra-individual variability in mean fixation time towards each of the two AOIs is considered, the *t*-test for dependent samples we performed showed no significant difference; *t*(26) = -1.712, *p* = 0.099.

For the results of the factorial analyses, we first noted that there was no visual preference for the mouth compared to the eyes’ regions in any of the three age groups (see intra-group difference in analysis b in Table 3). Children from the three different age groups (i.e. 11–24 months [*t*(5) = 1.967, *p* = 0.106], 25–36 months [*t*(11) = 0.472, *p* = 0.646] and 37–60 months [*t*(8) = 0.939, *p* = 0.375]) all looked at both AOIs equally: neither the differences in fixation times on each AOI between the three age groups (see inter-group difference in analysis b in Table 3), nor the interaction was significant, *F*(1, 24) = 0.674, *p* = 0.519.

**Table 3:** Mean (SD) fixation times in ms. as function of face areas, age group, residency location and SES.

	*N*	Fixation time (ms.)				Intra-group difference^1^	
		Mouth area		Eyes area		*t*	*p*
		*M*	(*SD*)	*M*	(*SD*)		
**(a) Total sample**	27	3906.80	(2187.18)	2546.53	(2056.86)	-1.712	0.099
**Factorial analysis**							
**(b) Age group**							
11–24 months	6	4871.63	(1931.77)	1841.13	(2040.49)	1.967	0.106
25–36 months	12	3519.87	(2425.77)	2907.65	(2124.48)	0.472	0.646
37–60 months	9	3779.51	(2045.24)	2535.30	(2091.07)	0.939	0.375
Inter-group difference^2^	*F*(1, 24) = 0.773, *p* = 0.473			*F*(1, 24) = 0.518, *p* = 0.602			
Interaction^3^	*F*(1, 24) = 0.674, *p* = 0.519						
**(c) Residency location**							
Rural areas	10	3269.54	(2016.65)	3350.49	(1952.20)	-0.066	0.949
Urban areas	17	4281.67	(2254.53)	2073.61	(2022.26)	2.195	0.043
Inter-group difference^4^	*t*(25) = 1.169, *p* = 0.253			*t*(25) = -1.604, *p* = 0.121			
Interaction^3^	*F*(1, 25) = 2.011, *p* = 0.169						
**(d) SES**							
LML	7	2715.83	(1033.48)	3799.81	(1942.74)	-0.984	0.363
HMH	20	4323.65	(2345.95)	2107.88	(1953.53)	2.357	0.029
Inter-group difference^2^	*t*(28) = -0.586, *p* = 0.562			*t*(28) = 1.028, *p* = 0.313			
Interaction^3^	*F*(1, 28) = 0.793, *p* = 0.381						

Results of T-tests and ANOVAs about intra- and inter-group differences and interactions are about intra-group difference were indicated in each condition.

^1^T-test for dependent samples; ^2^One-Way ANOVA; ^3^Mixed factorial ANOVA;^4^T-test for independent samples.

For the residency location’s factor, the results indicated that children living in rural areas did not show a visual preference for either of these two AOIs [*t*(9) = -0.066, *p* = 0.949], while those living in urban areas had a significantly higher fixation time on the mouth region (*M* = 4281.67, *SD* = 2254.53) than on the eyes’ region (*M* = 2073.61, *SD* = 2022.26), *t*(16) = 2.195, *p* = 0.043.

We observed similar results with the SES factor: only children from families with a medium–high to high SES looked significantly longer at the mouth area (*M* = 4323.65, *SD* = 2345.95) than at the eyes’ area (*M* = 2107.88, *SD* = 1953.53). No visual preference was observed in children from families with low to medium–low SES.

Finally, neither inter-group differences nor interactions tested were significant for the last two factors (see analyses c and d in Table 3).

Taken together, these results indicate that the AOIs in faces have an impact on the visual preference of the whole sample, suggesting a preference for the mouth region. However, this preference is not significant when we study the visual preferences within participants. In task 1, the results suggest that the type of movement has a significant impact on visual preferences, as well as when this preference is studied within-subjects. Specifically, our results suggest that when Ecuadorian preschoolers are presented with dynamic images of faces, there is a significant preference for the mouth region, but only in the whole group, an effect that does not appear when the fixation time between the two regions is compared within-subjects. On the contrary, when participants have the choice, they significantly prefer human movements against object movements, and this preference is maintained in the whole sample as well as within-subjects. Finally, in both tasks the developmental factor does not seem to have an impact, as no difference appears between age groups.

### Parental perceptions

We used the parent's perceptions as a measure of the feasibility of eye-tracking studies in Ecuador. 144 responses were analyzed. All caregivers reported a feeling of comfort during the experiment. Almost all participants (99%) reported they would agree to invite a friend or family member to participate in a similar study with their child. The question relative to caregivers’ questions or comments about the experiment was analyzed through Content Analysis, a method used to identify emerging categories from participants' answers^47,48^. The responses and comments to this open-ended question were transcribed verbatim and classified in at least one of the three emerging categories. The first category (10% of participants) was related to the way an eye-tracker functions (e.g., “How does it know where my child looks?", “How does it work?”). The second category (26% of participants) was related to the stimuli or about the calibration procedure, (e.g., “Why is there a child next to a wheel, “What do the points mean?”). The third category referred to an absence of questions (65% of participants). Interestingly, a homogenous sub-group of participants, from a rural area of residency and from families with low to middle-low SES (*n* = 17) did not ask any questions nor provide any comments.

## Discussion

The overarching aim of this study was to define the adaptability of the eye-tracking technique to semi-experimental and non-experimental settings in low- and middle-income contexts. To address this question, we used stimuli and procedures that have been previously used in occidental studies in order to quantify preferential visual attention towards socially salient stimuli. We also studied parents’ perceptions of the eye-tracking application.

The analyses of mean fixation times in task 1 showed a visual preference for human movements compared to object movements, whatever age, residency location or SES. This preference is maintained as well within-subjects confirming previous findings that used similar stimuli^21,22,49^.

The analyses of mean fixation times in task 2 showed that the AOIs on faces inner features have a significant impact on visual preference for the mouth’s region in the whole sample, but not when visual preference is studied within-subjects. These outcomes may be interpreted as a part of the mixed results reported by the literature. A first explanation to the variability of results related to the preference for the mouth’s region is the influence of the stimuli type and design used^13,50^.

An alternative explanation corresponds to complex developmental milestones. While some results indicate that two-year-olds with ASD look less at the eyes’ area and more at the mouth’s area compared to TD children^34^, others studies have found typical levels of looking times towards the eyes and reduced looking times towards the mouth in TD children aged 2 to 4^36^. In another study comparing ASD to TD preschoolers, Nakano et al.^51^ found that the majority of TD participants spontaneously directed their gaze towards the mouth of a person speaking. Based on this result, the authors suggested that this could be a necessary step in typical development. A finding that agrees with outcomes indicating that greater amounts of fixation time on the mother’s mouth during live interactions at the age of 6 months predicted higher levels of expressive language at the age of 18 months^52^. Alternative explanations to the preference for the mouth area, such as the size of the mouth and teeth of actors cannot be ruled out.

The analyses of the parental perception showed that eye-tracking setting is very well accepted and adapted. All the participants felt comfortable with the procedure during the experiment. Although 99% reported they would agree to invite a friend or family member to participate with their child, the rate of adherence explained by the presence of the researcher could not be ruled out. Parents' comments and interests were mostly related to the calibration procedure (dots moving around on the screen) and about the meaning of the stimuli. A small number of questions were related to the technology or the way eye-tracking works to capture the direction of the gaze. Interestingly an important proportion of participants did not provide comments or questions when given the opportunity. Most of the participants in this case belonged to the LML SES, suggesting that the presence of the experimenter could have had an influence.

The first limit of this study relates to the stimuli used in task 2. Some scenes may have shown more salient in certain features of the face, depending on the actors, that are difficult to control (for example the size of the mouth relative to the size of the eyes, size of the teeth, etc.) A high rate of calibration difficulties has been observed in this sample, suggesting the need of using a character who can more easily attract children's attention instead of calibration points. Visual acuity, which is a potential confounder, has also been difficult to control. The results of this study must be viewed with caution due to the small size of the sample. The current study was limited to one low- and middle-income setting; it is unlikely, however, that other understated elements not detected in this study would create major barriers for the implementation of eye-tracking procedures in a context such as Ecuador.

In spite of these limitations, this work provides some evidence on the feasibility of eye-tracking studies in typically developing young children in a low- and middle-income setting. It also suggests that a paradigm of visual preferences for human and non-human movements would be valid for use among the Ecuadorian population, in urban and rural areas, and in different socioeconomic contexts.

The study of social orientation in typical development, is necessary in order to provide a sound basis for explorations of atypical developments, such as those characterizing children with ASD. However, the use of shared protocols and experimental paradigms allowing data collection across different cultures and socio-economic settings would be necessary to determine whether the utility of eye-tracking technique extends beyond the mapping of socio-cognitive development, and could inform on its potential clinical utility in the identification of early markers in different socio-cultural contexts.

## Supplementary Information

Supplementary Video 1.

Supplementary Video 2.

Supplementary Video 3.

Supplementary Video 4.

Supplementary Video 5.

Supplementary Video 6.

Supplementary Video 7.

Supplementary Video 8.

Supplementary Video 9.

Supplementary Video 10.

Supplementary Video 11.

## Data Availability

The datasets generated and analysed during the current study are available from the corresponding author on reasonable request.
